# Feasibility of Full-Range Replacement of Natural Coarse Aggregates with Recycled Foam Concrete Aggregate: Effects on Rheology, Mechanical Degradation, and Shear Resistance

**DOI:** 10.3390/ma19081622

**Published:** 2026-04-17

**Authors:** Huan Liu, Xiaoyuan Fan, Alipujiang Jierula, Tian Tan, Yuhao Zhou, Nuerlanbaike Abudujiapaer

**Affiliations:** 1 College of Civil Engineering and Architecture, Xinjiang University, Urumqi 830017, China; liuhuan@xju.edu.cn (H.L.); fanxiaoyuan@stu.xju.edu.cn (X.F.); zhouyuhao@stu.xju.edu.cn (Y.Z.); nurlanbike@stu.xju.edu.cn (N.A.); 2 Xinjiang Key Laboratory of Building Structure and Earthquake Resistance, Xinjiang University, Urumqi 830017, China; 3 CCCC-SHEC Fifth Engineering Co., Ltd., Xi’an 710119, China

**Keywords:** recycled foam concrete aggregate, rheology, mechanical degradation, shear resistance

## Abstract

The urgent global need for sustainable infrastructure drives the demand for high-value buildings and waste removal. This paper studies the feasibility of using recycled foam concrete aggregate (FCA) as a substitute for natural coarse aggregate (NCA) in concrete and studies its impact on rheology, mechanical degradation, shear resistance, and the full-range replacement ratio (0–100). The experimental results show that the monotonic change in the workability of fresh concrete determines the lubrication threshold at 60% replacement, which is driven by the volume proportion effect. Beyond this value, capillary suction dominates, and the viscosity rises rapidly. From a mechanical perspective, the porous structure of FCA is conducive to “internal curing” so that moisture is released from the drying interface, but it also becomes a source of defects that change the fault topology. Specifically, the critical transition of the shear failure mode shifts from the debonding of the interface to the crushing of the cross-particle aggregate. At this time, the shear capacity decreases substantially, experiencing a reduction of 71.8% when completely replaced. There is a strong correlation between ultrasonic pulse velocity (UPV), rebound number, and compressive strength, and a multivariate nonlinear regression model (R^2^ > 0.85) with non-destructive strength prediction is ultimately obtained. Based on the balance between mechanical capacity and resource cyclability, an optimal alternative zone of 20% to 40% is proposed. This work not only provides a mechanism for multi-scale coupling between pore structure and structural properties but also provides a data-driven method for the safety assessment of lightweight recycled aggregate concrete (RAC).

## 1. Introduction

At present, the unified global task of ecological management and sustainable resource governance is accelerating the transformation of the construction industry to a cycle-driven structure [1]. According to this trajectory, the high-level national blueprint has established a comprehensive framework for green buildings and clearly proposed the high-value addition of construction and demolition waste [2]. These regulatory directives have a dual-purpose mechanism, which not only reduces the pressure of the imminent exhaustion of virgin aggregates but also promotes the separation of key issues such as infrastructure expansion, environmental degradation, and excessive resource consumption [3]. In addition, the strengthening of the localised low-carbon program makes the transition to lightweight functional materials very important, especially relying on the recycling of foam concrete [4,5]. Under the global strategic framework of the United Nations Sustainable Development Goals (SDGs) [6], the necessity of resource optimisation and closed-loop waste treatment has become a guiding principle for pioneering research and development measures [7]. Although the technology of recycled aggregate concrete (RAC) has made great progress under the promotion of the policy, there are still fundamental obstacles to integrating recycled foam concrete aggregate (FCA) into mainstream structural applications [8]. Because FCA, itself, has complex material characteristics, especially the obvious randomness of its material properties, the path of mechanical degradation in the concrete matrix is elusive, and there is generally a lack of evidence of its long-term durability. Therefore, it is crucial to bridge the above-mentioned key gaps to turn FCA from a hidden waste flow into a viable high-performance structural part [9]. When looking for sustainable infrastructure, there is an implicit requirement for a comprehensive review of FCA; this commitment is a critical pathway to decouple construction growth from environmental degradation.

In addition, this study has a double meaning. One is to provide a basic empirical basis for improving the conceptual framework of recycling cement composite materials, and the other is to become a catalyst for the added commercial value of construction waste [10]. The purpose of this study is to explore the evolution of the behaviour of porous recycled aggregates under complex load conditions. One of the main purposes is to determine the basic coupling mechanism between macroscopic mechanical properties and non-destructive testing parameters, then promote an in-depth understanding of the random damage propagation law of dominant heterogeneous materials [11]. From a practical engineering standpoint, the research results provide an important technical basis for formulating standards for sustainable building materials and evaluating the reliability of service infrastructure [12]. By analysing the differences between the research of bridging theory and the application of industrial policies, this study provides engineering guidance for construction and demolition waste recycling to become high-quality resources, thereby promoting the transformation of the structure of the ecologically efficient construction sector [13].

According to the existing research framework, this survey mainly describes the various impact mechanisms of waste FCA on the concrete matrix [14]. The main purpose of this study is to reveal the synergistic relationship between FCA integration and physical and mechanical response and to establish the correlation between non-destructive evaluation (NDT) characteristics and the basic shear performance of structural components [15]. The research trajectory must be carried out strictly according to the four core scientific issues clarified herein, namely:

(1) Macroscopic rheological and mechanical evolutions: Describe the nonlinear effect of FCA on the workability and hardening properties of fresh concrete instead of comparison. It is necessary to create a quantitative response model to link the volume occupation effect of porous aggregates with the capillary suction effect and the evolutionary trajectory of decline and reduced compressive strength [16].
(2) Micro-mechanism and damage evolution: Investigation of the dual mechanism of FCA’s porous structure. The focus is on analysing the internal curing effect of the interface transition zone (ITZ) caused by the kinetics of water release [17] and on revealing the essence behind the transformation of the failure mode from interface debonding to cross-particle aggregate fracture [18].
(3) NDT framework: Verify the ultrasonic pulse velocity (UPV) and rebound number to reflect the development of pores and surface density inside FCA concrete, that is, characterise the changes in pores and surface density inside FCA concrete [19]. Another important purpose is to explain what the interaction mechanism between sound-wave propagation and structural integrity is and to establish a multivariable nonlinear regression model to accurately predict the intensity [20].
(4) Shear behaviour and resistance mechanisms: Investigate the impact of FCA integration on the final shear bearing capacity and fracture topology of concrete components. The focus is on the study of the dynamic process of aggregate interlock degradation and reducing the effective contact area to explain the main factors affecting the shear failure mode under different substitution ratios [21].

This research uses a new comprehensive way of arranging materials science and structural mechanics to distinguish itself. It is the first systematic explanation of the phenomenon of “internal curing” in FCA porous structures, as well as the dynamics of aggregate interlocking and reconfiguration. Filling the existing theoretical gap with respect to the multi-scale coupling between material properties and structural properties not only promotes basic understanding but also stipulates an optimised alternative FCA protocol [22]. The above results lay a solid foundation for the high-value addition of waste foam concrete, in addition to providing basic design guidelines [23]. Based on the principle of mechanical and NDT of porous media, the survey combines empirical verification with mechanical deconstruction, ultimately achieving the unity of performance optimisation and strict safety assessment of RAC [24].

## 2. Materials and Experimental Methodology

### 2.1. Characterisation of Raw Materials

Ordinary Portland cement (P·O 42.5, supplied by Xinjiang Tianshan Cement Co., Ltd., Urumqi Municipality, China) is the main hydraulic binder in this survey. Its physical properties—namely, fineness, setting time and compressive strength—all meet the current national regulatory standards. Natural river sand is used for fine aggregates, and its surface density is 2.62 $g/{cm}^{3}$ [25]. The physical characterisation gives a continuous particle size curve, a uniform particle size distribution, and a natural round shape [26]. Favourable properties that are beneficial to morphology can improve the packing density of the concrete matrix, thereby improving the macro-mechanical properties.

**Figure 1:**
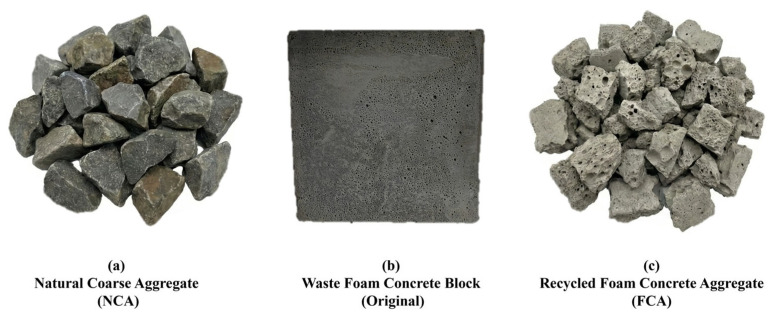
Macroscopic morphology of aggregates (sample sizes: 5–20 mm for NCA and FCA; 100 mm × 100 mm × 100 mm for the waste foam concrete block).

For the coarse fraction, natural pebbles continuously graded with 5–20 mm are used, the surface density is about 2.65 $g/{cm}^{3}$, and the water absorption rate is less than 1.0% [27]. The surface texture of these pebbles is characterised by medium roughness and flatness, which are conducive to the dense ITZ between aggregates and slurry [28]. The comparative macroscopic morphologies of the natural and recycled aggregates are presented in Figure 1. In order to ensure the rigour of the empirical evidence and the universality of the discovery, all raw materials signed a strict material selection agreement. Multiple sampling and testing show the consistency and representativeness of the material characteristics, which is a standardised workflow that can minimise the differences caused by quality and ensure the high repeatability of the research [29].

### 2.2. Fabrication Protocol for FCA

**Figure 2:**
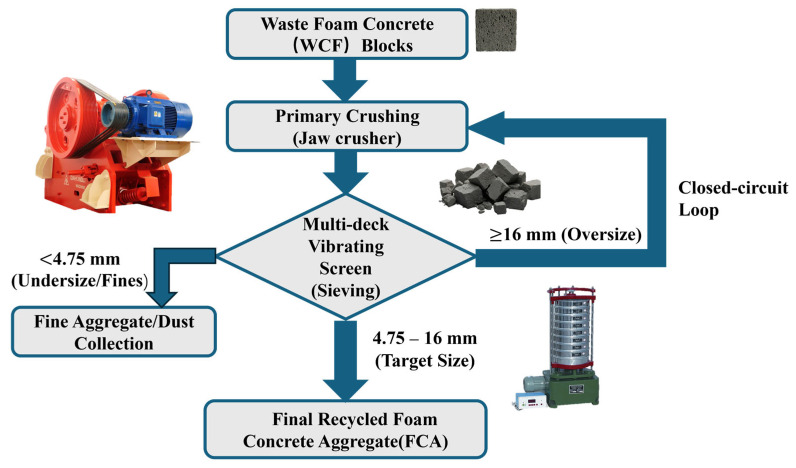
Preparation of recycled foam concrete aggregate.

The production of FCA is completed by a two-phase process characterised by mechanical crushing and multi-stage fractionation, as schematically illustrated in Figure 2.

#### 2.2.1. Controlled Comminution Phase

Waste foam concrete is treated with a closed-circuit jaw crusher (PE-250×400, Henan Liming Heavy Industry Science and Technology Co., Ltd., Zhengzhou, Henan, China). The key process parameters, especially the feeding rate, rotor speed, etc., are strictly controlled for the purposes of improving the geometry of the aggregate and reducing the accumulation of micro-damage inside the aggregate skeleton caused by mechanical stress [30].

#### 2.2.2. Precision Fractionation and Grading Control

**Figure 3:**
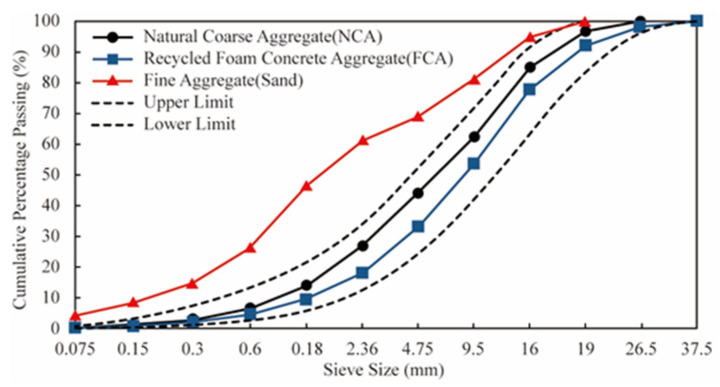
Particle size distribution curves.

The multi-layer shielding unit integrated with the high-frequency vibration system is used to classify the products. Through the correction of the mesh diameter and vibration amplitude, the effective particle size of FCA is strictly limited to between 4.75 and 16 mm, removing large particles and small particles. This process can ensure uniform particle size distribution (PSD), the curves of which are detailed in Figure 3, and preserve the physical integrity of recycled particles [31].

#### 2.2.3. Real-Time Monitoring and Quality Assurance

The whole manufacturing process should rely on the automatic data acquisition system to monitor the grinding energy consumption, screening efficiency, fine content and other process indicators in real time [32]. The process design not only meets the requirements of FCA particle integrity but can also accurately control PSD. Therefore, it can avoid the processing problems caused by fluctuations and provide reliable basic materials for the scientificity of subsequent experimental data [33].

### 2.3. Physico-Mechanical Profiling of FCA

We systematically evaluate the key physical index of FCA to explain the mechanism of its impact on the specific matrix.

#### 2.3.1. Bulk Density and Pore Characteristics

According to the standard tapping method, the volumetric density of FCA is between 1.20 and 1.35 g/cm^3^, which is significantly lower than that of natural coarse aggregates (NCA) [34]. The resulting decrease in density is entirely caused by its highly developed internal pore network. In the mixed design, this characteristic not only restricts the volume ratio of aggregates but also restricts the improvement of the volume stability of concrete. It must rely on the optimisation of the water–binder ratio (W/B) and the dose of high-efficiency superplasticizer for compensatory adjustment [35].

#### 2.3.2. Moisture Uptake and Hydrodynamics

After constant weight testing, the water absorption rate of FCA is 15–25%, which is far higher than that of traditional aggregates. The obvious hygroscopicity fundamentally changes the effective water–cement ratio and water migration behaviour in fresh concrete. Therefore, a compensation mechanism for extra water must be established [35]. In addition, the internal curing effect of the hardening stage should be taken into account to avoid the fluctuation of workability and interface drying caused by water competition [36].

#### 2.3.3. Crushing Value and Strength Boundaries

Crushing tests indicate that the crushing values of FCA range from 15% to 25%. Notably, the intrinsic strength of individual FCA particles surpasses that of the macroscopic parent foam concrete blocks. This phenomenon is primarily attributed to the “selective crushing” effect during mechanical processing: the most porous and fragile regions of the original concrete are pulverized into fines, while the relatively dense and structurally sound fragments are retained as aggregates. Furthermore, as noted in Reference [36], the micro-pore structure of FCA facilitates an “internal curing” mechanism that optimises the surrounding matrix, although its overall cracking resistance remains limited compared to natural aggregates [37]. The index defines the maximum bearing capacity of FCA concrete as an important parameter for the construction of mechanical interpretation frameworks and strength prediction models.

### 2.4. Mix Proportion Design and Optimisation Strategy

#### 2.4.1. Mix Proportioning and Variable Isolation

This study strictly follows the principle of concrete hybrid design and systematically analyses the evolutionary law of matrix properties under different FCA dosage conditions. The FCA replacement rate ($R_{FCA}$) is designated as the intermediate variable of the main component analysis. Using the equivalent volume substitution method, six discrete gradient levels are obtained—namely, 0%, 20%, 40%, 60%, 80% and 100%—forming an experimental matrix containing the entire dose spectrum [38]. In order to ensure the comparability of each group, all combination ratios are calibrated according to the current national and industry standards. During the variable control period, the dosage, sand ratio and total aggregate volume of the cement material remain unchanged, and the target variables are isolated from external interference. The gradient layout not only illustrates the relationship between the microstructure and macro-mechanical evolution but also provides systematic data support for the establishment of the coupling relationship between mechanical indexes and NDT parameters [39]. This plan also takes into account the requirements of resource regeneration efficiency and structural stability, giving a new theoretical perspective to the material engineering application of green buildings [40].

#### 2.4.2. Optimisation Strategy and Moisture Regulation

In order to quantitatively characterise the role of FCA, this paper strictly abides by the principle of a constant water–binder (W/B) ratio and separates its influence and matrix performance. The design logic is described as follows:

(1) Variable control principle: With the increase in FCA’s volume replacement, the volume of NCA also decreases proportionally. The cement content, fine aggregate ratio and total aggregate volume remain unchanged, excluding interference from other components [41].

(2) Moisture compensation mechanism: Given the porous nature of FCA, additional water ($W_{ad}$) is introduced as a regulating variable. By monitoring the moisture uptake kinetics, pre-wetting water is precisely compensated for to offset the negative impact of FCA absorption on fresh concrete fluidity, ensuring consistent slump levels across all batches [42].

(3) The effective W/B ratio is fine-tuned, and the mixing parameters are dynamically adjusted according to the effective water obtained after absorption correction. The strategy adequately reflects the intrinsic impact of polymer absorption on ITZ and polymer compatibility and provides a statistical sample that can be compared for subsequent intensity modelling and NDT correlation [43].

#### 2.4.3. Scientific Rationale and Logical Framework

Therefore, this design takes a rigorous logic system as the starting point to determine the scheme. Due to the limitations of standard preparation and curing conditions, the full-spectrum replacement gradient (0–100%) successfully separates the change in the overall composition from the evolution of the attribute. The system has three advantages, namely:

(1) Data robustness: Standardized variable control ensures statistical reproducibility and representativeness.
(2) Mechanistic correlation: A uniform control environment provides a reliable physical platform for analysing the synergistic effects of FCA porosity and ITZ characteristics on mechanical behaviour and NDT response [44].
(3) Combination of theory and practice: This plan not only deepens the theoretical understanding of green material optimisation but also gives a foundation of common understanding for the study of shear degradation, NDT model and safety evaluation, and other related issues [37].

### 2.5. Specimen Preparation and Curing Regimen

#### 2.5.1. Mixing Protocol and Casting Procedure

Current national standards are strictly executed to ensure high scientific representativeness. Raw materials are precisely weighed according to the preset matrix ($R_{FCA}$ = 0–100%) [33]. All component pre-screening and pretreatment ensured grading continuity and impurity control. The forced mechanical stirring method is used to strictly control the feeding order and mixing time to ensure uniformity. The mixture is cast, put it into a standard mould, and compacted on a high-frequency vibrating table [45]. The vibration frequency and amplitude are dynamically adjusted according to the rheological characteristics of different levels so as to reduce trapped air as much as possible and ensure the desired density [46].

#### 2.5.2. Standard Curing Regime

To promote hydration and micro-structural densification, a strict curing protocol is mandated:

(1) Environmental control: Specimens are demoulded after 24 h and immediately transferred to a standard curing room (temperature: 20±2 °C; relative humidity ≥ 95%).
(2) Curing duration: Continuous curing is maintained until the 28-day age. This prevents premature moisture loss, ensuring the orderly evolution of hydration products and stable strength development [47].
(3) Systematic error elimination: The uniform environment eliminates non-uniform shrinkage and strength variance caused by ambient fluctuations, providing a consistent baseline for NDT and mechanical analysis [36].

#### 2.5.3. Quality Assurance and Technical Mitigation

The following steps are taken to address specific challenges in FCA concrete preparation:

(1) Density homogeneity: Vibration parameters are optimised to prevent under-compaction or segregation-induced stress concentrations caused by the rheological changes associated with porous FCA [48].
(2) Environmental stability: An automated multi-point monitoring system regulates the curing environment to prevent micro-cracking induced by uneven evaporation [49].
(3) Geometric precision: High-precision 3D measurement tools verify specimen dimensions, strictly controlling tolerances (e.g., perpendicularity and planeness) to ensure data validity [50].

### 2.6. Methodology and Multi-Dimensional Evaluation System

A complete evaluation system is created, including macroscopic mechanics, fresh workability, micro-structural integrity and shear behaviour. All tests are carried out in accordance with national and international standards to ensure the accuracy and comparability of the results.

#### 2.6.1. Rheological Characterisation of Fresh Concrete

A standard depression test is used, including a calibrated depression cone (Cangzhou Road and Bridge Instrument Co., Ltd., Cangzhou, China) and high-precision measuring instrument (1 mm resolution), to evaluate the fluidity and uniformity. The procedure is strictly implemented in accordance with GB/T 50080-2016 [51]. In order to offset the environmental interference, the test is performed under a temperature of 20±2 °C with a humidity of 60%. This indicator is used to measure whether the project can be built [52].

#### 2.6.2. Mechanical Properties and NDT

**Figure 4:**
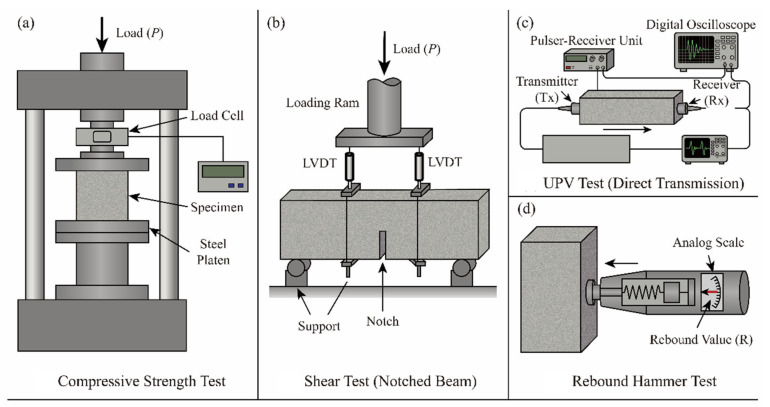
Schematics of experimental test setups: (**a**) uniaxial compressive strength test; (**b**) shear test using notched beam; (**c**) UPV measurement; (**d**) rebound hammer test.

The testing process is described as follows:

(1) Single-axis compression loading is performed using a universal testing machine controlled by a 3000 kN servo control on a 100 mm cube to obtain a stable stress–strain curve, and the loading rate is 0.1 MPa/s (i.e., per GB/T 50081-2019 [53]) [54].
(2) UPV, according to the provisions of ASTM C597, uses a 54 kHz high-frequency pulse transmitter (ZBL-U520, Beijing ZBL Science & Technology Corp., Ltd., Beijing, China) to measure the internal structure [55]. Wave velocity analysis can quantitatively characterise the distribution of defects and changes in pores in the FCA matrix.
(3) Rebound number: A calibrated digital rebound hammer (HT-225W, Jinan Langrui Detection Technology Co., Ltd., Jinan, China) (per GB/T 50311-2016 [56]) assesses surface density and uniformity, providing cross-verification with UPV data [57]. The comprehensive experimental configurations for both mechanical and non-destructive testing are conceptualized in Figure 4.

#### 2.6.3. Analysis of Shear Behaviour and Fracture Mechanism

In order to evaluate the contribution of FCA to structural performance, a purposeful shear test is set up. In the displacement control mode (0.2 mm/min), the servo shear system is used to test the notched beam specimens (150 mm × 150 mm × 300 mm) with four-point bending support [58]. High-precision LVDT records the load and displacement curves synchronously with the weighing sensor. The failure standard is expressed by the peak load, plus crack expansion and fracture surface analysis [59]. Each configuration should have at least three replicas of specimens, and the instrument should be systematically calibrated regularly. The purpose of this experiment is to understand the adhesion of the interface and its impact on the shear ability through testing and to provide key data for the analysis of the mechanism of polymer interlock and interface failure [60].

## 3. Results and Discussion

### 3.1. Evolutionary Impact of FCA Replacement Ratio on Fresh Concrete Workability and Mechanism Analysis

#### 3.1.1. Experimental Context and Characterisation Objectives

This section starts with the systematic study of the impact of the replacement rate of FCA on the sluggish evolution of newly mixed concrete. The essence of rheological variation is the logic of the flow capacity and cohesion of the mixture under the action of shear force and the control of the redistribution of water between paste and aggregates [11]. Due to the high porosity and low volume density of FCA, it will not only change the appearance volume of the matrix after it is added but also destroy the lubrication effect of the slurry due to the complex water adsorption and desorption process [15]. Using 0%, 20%, 40%, 60%, 80% and 100% test sample gradients, the main purpose of this study is to quantitatively uncover the mechanical effect of FCA on workability and lay the foundation for the comprehensive performance evaluation of RAC [61].

#### 3.1.2. Evolutionary Characteristics of Slump and Mechanism Analysis

**Figure 5:**
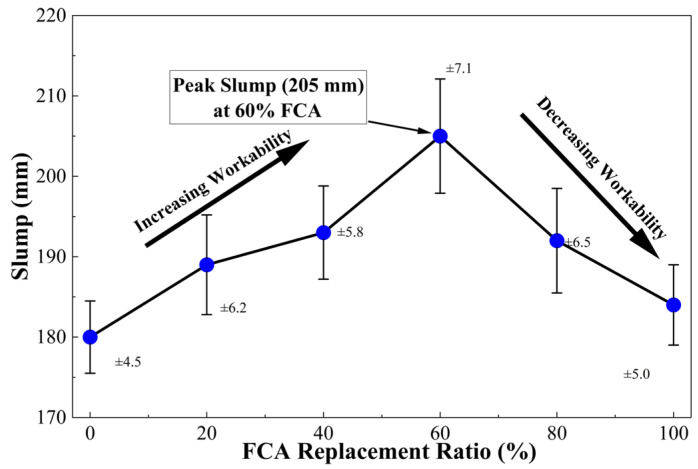
Effect of FCA replacement ratio on slump.

The empirical data plotted in Figure 5 reveals that slump exhibits a non-linear trend of “initial increase followed by a decline” as $R_{a}$ increases, peaking at 205 mm when $R_{a}$ = 60%, which underscores the unique regulatory law FCA imposes on concrete rheology. In the $R_{a}\leq$ 60 phase, the slump rises steadily from the baseline of 180 mm; this phenomenon is mechanically attributed to the “volume occupation effect” and “friction reduction effect” of FCA. Because the density of FCA is much lower than that of natural aggregates, the equivalent mass replacement increases the total volume of the mixture, thereby increasing the lubrication margin of the paste [15]. At the same time, the morphology of FCA particles can improve the shear flow between particles at medium and low replacement rates.

**Figure 6:**
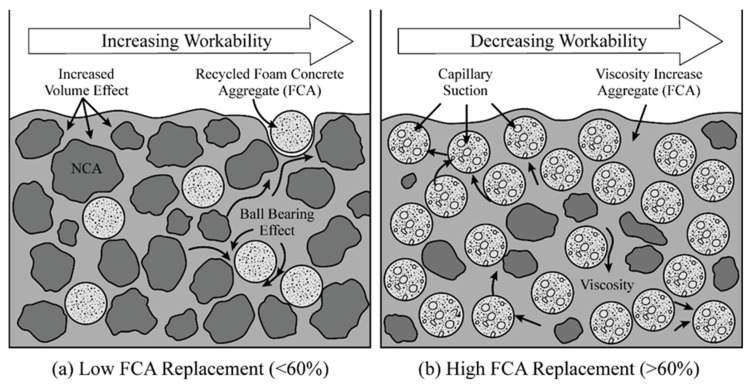
Mechanism of workability evolution.

However, once $R_{a}$ exceeds 60%, the slump begins a marked regression, dropping to 184 mm at 100% replacement. This inflection point signals that the “capillary suction effect” of FCA has begun to dominate system performance. In this high-replacement interval, the highly developed internal pore structure of FCA rapidly scavenges free water during the initial mixing phase. This causes an instantaneous drop in the effective water–cement ratio ($\omega /c_{eff}$) and a sharp surge in the apparent viscosity of the paste, thereby inhibiting the macroscopic deformability of the concrete [62]. Figure 6 conceptually illustrates the dynamic competition between these physical volume effects, and moisture migration mechanisms constitute the scientific essence of slump evolution in FCA concrete.

#### 3.1.3. Scientific Evaluation and Engineering Recommendations

From the comparative analysis of domestic and foreign literature, it can be seen that the fluctuation pattern described here is a unique physical structural feature of FCA that distinguishes it from the monotonous decline caused by the high absorption caused by the attached old mortar in traditional recycled aggregates [11,63]. FCA provides a new way of thinking to improve the constructability of lightweight concrete by adjusting the proportion of aggregates for the “compensation effect” that can be operated within a specific replacement range [23]. Since the study reported here includes strict control over the quality of raw materials and the test environment, the experimental results are very reliable [64].

From an engineering perspective, to achieve excellent liquidity, the positive gain feature of FCA can be used to adjust the mixing ratio. On the contrary, in order to reduce the loss of fluidity caused by the high replacement rate, it is necessary to accurately control the pre-saturation state of the aggregate or add chemical additives to achieve the purpose of rheological compensation [65]. The comprehensive evaluation of the impact of FCA on the comprehensive service performance of concrete should be combined with the subsequent analysis of mechanical strength and microstructure.

### 3.2. Multi-Scale Pore Characteristics of FCA and the Internal Curing Mechanism

#### 3.2.1. Multi-Scale Pore Structure and Moisture Transport Kinetics

FCA is characterised by a typical multi-scale stratified pore structure. Its internal porosity is composed of an interconnected three-dimensional topological network composed of nanometre-scale capillaries and micro-scale macropores so that FCA has a high specific surface area [66]. It can be seen from the porous medium theory that the structure determined both the lightness of FCA and the direction of water movement in the mixture [67]. At the beginning of mixing, FCA relies on considerable capillary suction to quickly suck in and store the mixed water, controlling the percentage of free water in the paste.

From the data, it can be seen that the abnormally small increase of 60% resulting from the “volume occupation effect” is caused by the decrease in FCA volume density. From the mechanical point of view, it is the temporary moisture storage mechanism of FCA pores that delays the thickening of the paste, thereby improving the microfluidity in this range.

#### 3.2.2. Evolutionary Mechanism of “Internal Curing” Based on Moisture Release

**Figure 7:**
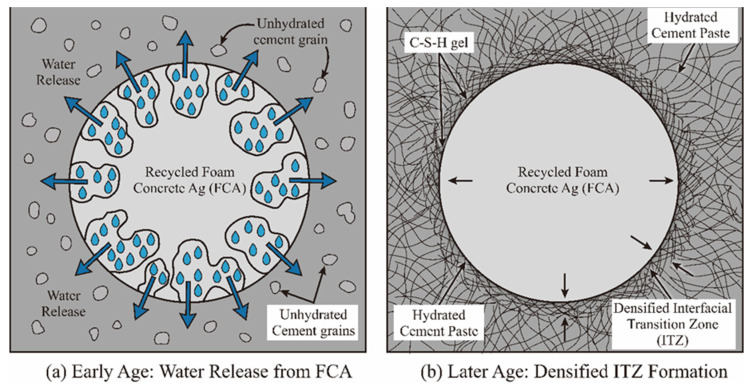
Schematic of the internal curing mechanism in FCA concrete: (**a**) early age—water release from FCA; (**b**) later age—densified ITZ formation.

The porosity of FCA offers great potential with respect to “internal curing” in concrete. The core of this mechanism is the process of the gradual release of water caused by chemical potential gradients [36]. With the hydration of cement and the decrease in the internal relative humidity, the water stored in the FCA pores begins to dynamically compensate for the surrounding water-consuming matrix. This water release method prolongs the hydration time window of cement particles and ensures the dynamic balance of the internal humidity of the matrix [68]. This internal curing mechanism is diagrammed in Figure 7.

From the micro-favourable position, the internal curing effect is conducive to improving the moisture environment of the ITZ and inhibiting the generation and development of microcracks. Theoretically speaking, this improves the overall continuity and density of the concrete structure. The size of this effect is related to the FCA replacement rate and pore saturation, which are important variables regulating the performance of recycled concrete [69].

#### 3.2.3. Engineering Significance and Research Prospects in Resource Circularity

The goal is to achieve a comprehensive understanding of FCA’s moisture regulation mechanism and its profound theoretical significance and practical value in the fields of the development of green building materials and resource recycling. This mechanism provides a scientific basis for optimising the mixing design of recycled concrete (such as precise adjustment of the water–binder ratio) and reducing the risk of early cracking of high-performance concrete [61]. In particular, it has obvious advantages in terms of improving the crack resistance of high-performance concrete, which can improve performance to a large extent [70].

In the future, it is necessary to improve the moisture absorption and heat dissipation efficiency of FCA to improve the long-term service performance of recycled concrete. Relevant strategies can focus on the improvement of pore structure and composite curing technology [66]. At the same time, to solve the problem of coupling between the internal water migration mechanism of FCA and structural evolution, it is necessary to use high-precision characterisation methods to quantitatively analyse multiple scales. This will deepen the understanding of internal curing dynamics and propel the engineering application of recycled foam concrete aggregates within high-performance green building systems [71].

### 3.3. Evolutionary Influence of FCA on Compressive Strength

#### 3.3.1. Experimental Protocols and Evaluation Benchmarks

This part systematically analyses the regulation law of concrete compressive strength as a function of FCA’s replacement rate ($R_{FCA}$). As an authoritative mechanical parameter to evaluate the bearing capacity and structural safety of concrete, the compressive strength trajectory is not only the basis for determining the application level of recycled aggregates [72] but also the quantitative standard for mixing and optimisation. In order to eliminate the impact of strain-rate sensitivity on strength determination, in strict accordance with international and national standards, in the experimental procedure, a 100-mm cube sample loaded with a precise control rate of 0.1 MPa/s is used [73]. In addition, in order to ensure the consistency of cement hydration, in the standard environment (temperature of 202 °C and relative humidity of 95%), all samples are cured at the same time point (28 d). A gradient test matrix with a replacement ratio of 0–100% is used to study the changes in characteristic strength and internal properties when FCA merging causes structural weakening.

#### 3.3.2. Characteristics of Strength Degradation and Non-Linear Regression Modelling

The experimental results show that that with the increase in the FCA replacement rate, there is an obvious nonlinear attenuation curve in the compressive strength. After 28 days, the intensity drops from the original 38.58 MPa to 22.36 MPa from the original 0% to 100%, accounting for 42.1% of the total output. The degradation trajectory can be divided into two parts:

(1) Mild degradation phase ($R_{a}\leq$ 40%): In this regime, strength loss is relatively contained (within 10%), suggesting that low-dosage FCA exerts a limited negative impact on the rigid skeleton of the matrix.
(2) Accelerated decay phase ($R_{a}>$ 40%): When the replacement rate reaches more than 40%, the intensity decreases rapidly, and in the range of 40–60%, the decline rate accelerates significantly. It can be seen that the structural defects caused by porous aggregates play a major role.

The selection of this specific functional form followed a comparative assessment of linear, exponential and power-law formulations. To mathematize this degradation logic, a non-linear regression framework based on $R_{a}$ was constructed. The rationale for this specific functional form is based on the observed two-stage degradation—initial stability followed by accelerated decline [74], which a standard linear model cannot capture. This form acts as a damage-evolution model, where ${\left(1-b\cdot R_{a}\right)}^{c}$ represents the strength reduction factor relative to the baseline strength a. Similar power-law frameworks for recycled aggregate concrete have been validated in previous studies [75]:

(1) $$\begin{array}{c} f_{c}=a{\left(1-b\cdot R_{a}\right)}^{c} \end{array}$$

In this formula, $f_{c}$ is the compressive strength (MPa), and $R_{a}$ is the FCA replacement ratio (%). The parameter expressed as a represents the baseline compressive strength of the control group (0% FCA), with a fitted value of 38.96 MPa. The terms b and c are dimensionless fitting coefficients that dictate the rate and curvature of strength degradation, with fitted values of 0.0057 and 0.68, respectively.

**Figure 8:**
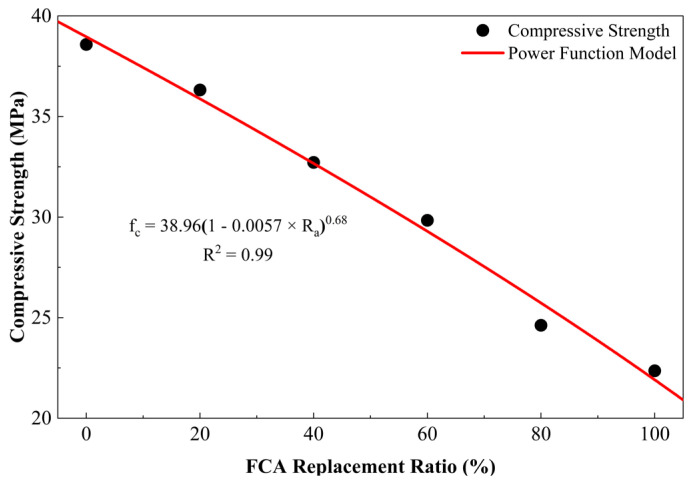
Non-linear regression of compressive strength vs. FCA replacement ratio.

While a simple linear regression model provides a reasonable approximation ($R^{2}\approx \;0.91$), it fails to account for the non-constant rate of strength loss observed in the experimental data. However, fitting analysis, as depicted in Figure 8, confirms that the proposed power-function model accurately captures the evolutionary characteristics of an initial plateau followed by accelerated decline ($R^{2}$ > 0.99, p < 0.1). The non-linear approach is statistically superior and physically consistent with the transition from interfacial debonding to accelerated aggregate crushing. The regression coefficients indicate that for every 10% increment in the replacement ratio, the average strength deterioration ranges between 3.5% and 5%, providing robust mathematical support for engineering strength predictions of FCA concrete.

#### 3.3.3. Macroscopic Failure Topologies and Mechanistic Dissection

**Figure 9:**
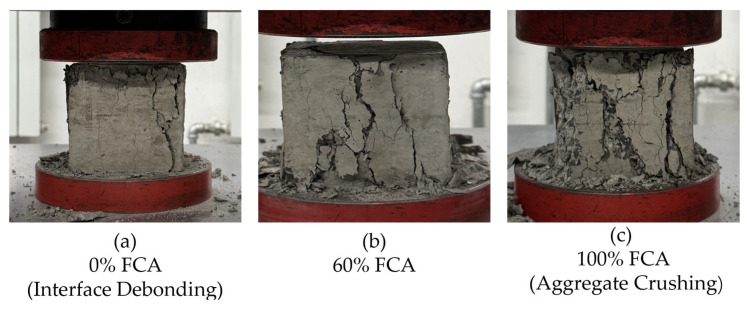
Failure patterns under compression with different FCA replacement ratios: (**a**) 0% FCA (Interface Debonding); (**b**) 60% FCA; (**c**) 100% FCA (Aggregate Crushing). (Specimen size: 100 mm ×100 mm × 100 mm).

The macromorphology of the failed specimen provides intuitive physical evidence through the measured strength reduction mechanism. Experimental observation shows that after the increase, the failure mode changes significantly from the original interface failure to aggregation failure, as represented by typical failure patterns in Figure 9. In the baseline group and the low-replacement group ($R_{a}\leq$ 20%), the main failure is the typical debonding of the old and new mortar interfaces. The main cracks extend to the gathering place and make a crack sound. The natural polymerisation intensity is still greater than that of the paste matrix, and the ITZ is the weak link of compression performance [76]. When it reaches more than 40%, a qualitative change will occur; the crack will no longer bypass the aggregate but will show an obvious cross-particle fracture effect.

With the cross-section containing 80% high-replacement specimens, it can be seen that a large number of FCA particles are directly sheared or crushed. The change from “interface peeling” to “aggregate splitting” reflects the mode of compressive strength degradation.

The micron-level pores developed internally by FCA are not only moisture-regulating units but also the “defect source” of stress concentration [76]. The fragile hole wall cannot provide sufficient support stiffness under the action of external load, causing the crack to penetrate the aggregate directly. This leads to the “polymerisation weak link effect”, and the interface bonding performance is weakened, which is the main factor limiting the overall bearing capacity of high-replacement FCA concrete [77].

#### 3.3.4. Engineering Implications and Optimisation Strategies

Predicated on a balanced analysis of workability, mechanical competence, shear reliability and resource circularity, this study posits that $R_{a}\leq$ 40% constitutes the safety threshold for ensuring the reliability of FCA concrete structures. In this range, the material achieves peak workability while maintaining a residual strength of about 34.72 MPa, which meets the bearing-capacity requirements of the standard C30 structure. However, when the replacement rate exceeds 60%, remedial performance compensation measures need to be taken. Supplementary cementitious materials, such as slag or silica fume, can be added to strengthen the paste matrix, or aggregate strengthening technology (polymer impregnation, carbonation modification, etc.) can be used to improve the stiffness of the FCA’s inner pore wall [78]. The quantitative evaluation and mechanistic analysis of this chapter not only deepen the understanding of the multi-scale mechanical behaviour of recycled foam concrete but also provide a theoretical basis for the relationship between later shear performance and non-destructive test characteristics [71].

### 3.4. Influence of FCA Replacement Ratio on Shear Performance and Mechanistic Dissection

#### 3.4.1. Theoretical Mapping of Aggregate Strength to Shear Interlocking Effects

The aggregate strength is the physical parameter that determines the shear ability of concrete, which directly affects the friction dissipation and geometric interlock effect in the matrix. Unlike the characteristics of NCA with dense minerals and high mechanical strength, FCA has obvious porosity and internal structural vulnerability [79]. From the perspective of material mechanics, the hard surface texture of natural aggregates is conducive to generating a large amount of mechanical locking resistance to crack propagation. The internal multi-scale residual pore structure of FCA greatly reduces its rigidity and deformation resistance, and contact stiffness is greatly reduced under the action of shear load. Such a difference in physical properties indicates that the fundamental paradigm of the internal stress transfer trajectory of concrete has changed from the original aggregation interlock-based concrete to the later FCA, which is dominated by matrix/interface weakening [80].

#### 3.4.2. Evolution of Shear Capacity and Cross-Sectional Weakening Mechanisms

**Figure 10:**
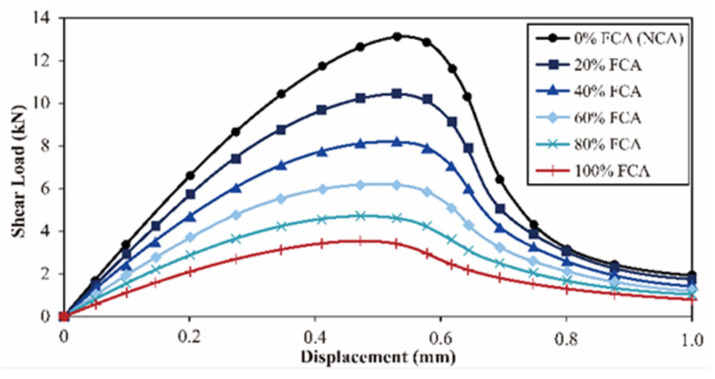
Shear load–displacement curves of concrete specimens with varying FCA replacement ratios.

The empirical dataset quantitatively unmasks a drastic attenuation in shear performance as the FCA replacement ratio ($R_{a}$) escalates. As $R_{a}$ increases from 0% to 100%, the ultimate shear capacity of the concrete suffers a monotonic diminution from 13.1 kN to 3.7 kN, as evidenced by the load–displacement curves in Figure 10. In particular, the cumulative reduction in shear strength (71.8%) obviously overshadows the changes in compressive strength at the same age [81]. It can be seen that shear kinematics enhance the inclusion of porous aggregates.

In terms of the micro-mechanism, FCA is easily affected by local fragmentation, micro-cracks, etc., in the stress concentration area. As a result, the effective contact area on the shear plane is greatly reduced. With the increase in the replacement ratio, the interlocking force between aggregates decreases exponentially due to particle fracture, and a reduction in the friction coefficient will cause frequent sliding and instability between aggregates. The loss of the interlocking force system will destroy the continuity of shear load transmission, resulting in rapid declines in shear stiffness and load-bearing capacity [82].

#### 3.4.3. Interface Evolution, Failure Modes, and Optimisation Strategies

**Figure 11:**
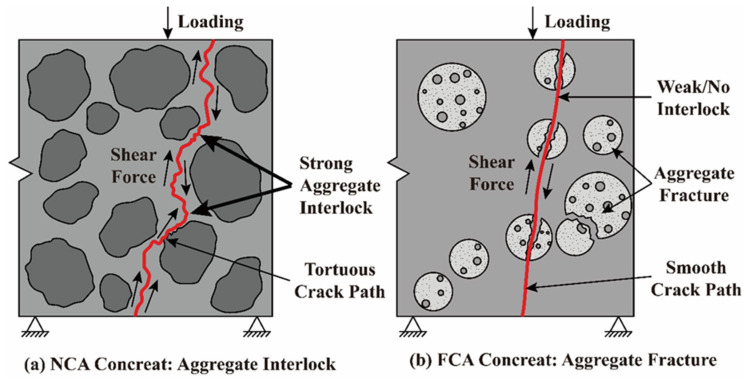
Schematic comparison of shear resistance mechanisms: (**a**) NCA concrete—aggregate interlock; (**b**) FCA concrete—aggregate fracture.

The residual old mortar attached to the surface of FCA, together with its unique porous topology, fundamentally changes the mode of energy dissipation during shear failure. In the ITZ, the interface layer yields in advance under the action of shear stress due to high porosity [83]. Therefore, the failure mode changes from the typical large-area “interface debonding/aggregate bypass” of natural aggregate concrete to “aggregate penetration/cross-particle splitting” observed in FCA concrete, a mechanistic contrast highlighted in Figure 11.

Enhancement of the elastic–plastic deformation characteristics leads to the uncontrolled consumption of energy during the loading process, resulting in early cracking and rapid expansion. Since FCA replacement has a huge adverse impact on shear reliability, it is essential to carefully evaluate structural safety for engineering applications with high replacement rates. In order to reduce the risk associated with aggregate interlock loss, future research should pay attention to the application of composite aggregate technology. A combination of natural aggregates and FCA is recommended to improve the gradient skeleton; alternatively, modifying the surface of FCA by polymer impregnation can improve its contact stiffness [84] so as to achieve the effective use of green building materials and ensure the safety of structural shear.

### 3.5. NDT Characteristics and Strength Prediction Modelling of FCA Concrete

#### 3.5.1. UPV: Internal Pore Evolution and Acoustic Transmission Mechanisms

**Figure 12:**
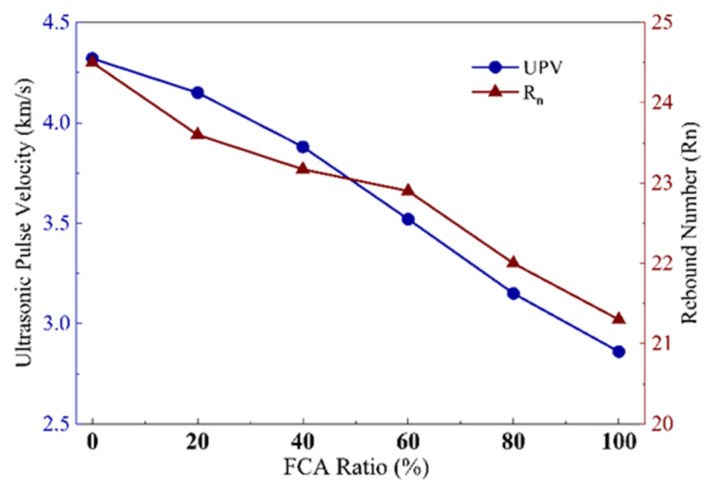
Variation of UPV and Rebound Number ($R_{n}$) with different FCA replacement ratios.

UPV is an important non-destructive indicator for evaluating the integrity of concrete structures. It reflects the influence of the elastic modulus and density of the medium on the propagation dynamics. As plotted in Figure 12, when the FCA replacement ratio ($R_{a}$) increases, UPV undergoes obvious nonlinear attenuation: the average speed value drops from 4.32 km/s under 0% replacement to 2.86 km/s under 100% replacement. This degeneration feature adequately reflects the deterioration of the internal structure caused by FCA merging.

**Figure 13:**
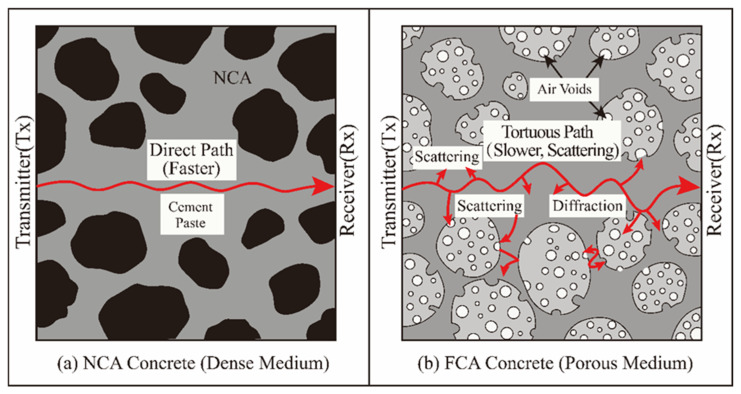
Schematic model of UPV propagation paths: (**a**) NCA concrete (dense medium); (**b**) FCA concrete (porous medium).

From the perspective of acoustic scattering theory, the complex network structure formed by nanometre- to micron-scale pores inside FCA constitutes complex acoustic discontinuity. These interfaces will cause serious waveform conversion and energy consumption. With increasing porosity, the effective propagation route of sound waves becomes very tortuous (see the propagation path model in Figure 13), and the appearance speed decreases rapidly.

In addition, there is a difference in acoustic impedance between the porous aggregate and the cement matrix, resulting in microcracks, which further reduce the transmission efficiency [84]. Quantitative analysis shows that there is a high statistical correlation between the attenuation rate of UPV and the increase in porosity, which proves that UPV can be used as a sensitive physical indicator to monitor the development trend of the relaxation of the FCA concrete microstructure.

#### 3.5.2. Rebound Number and Surface Hardness: Mapping Logic of Surface Density and Aggregate Features

The rebound value ($R_{n}$) is the metric for measuring the surface hardness and local stiffness of concrete. The surface-sampling results of different systems show that the average rebound value exhibits a gradual downward trend, not a sharp downward trend, from 24.0 in the control group to 21.3 in the complete replacement group. Unlike for UPV, the attenuation is less pronounced, indicating that the sensitivity of surface hardness to porous aggregate inclusions is lower than that of internal acoustic parameters.

The physical reason is the filling effect of mortar. Near the surface, it can partially offset the effect of porous aggregates, causing a decline in stiffness. However, under the high-replacement system ($R_{a}\geq 80\%$), the probability of large-aperture holes near the surface increases, which significantly increases the discreteness of the rebound value, indicating that the heterogeneity of the surface hardness has increased. Linear regression [84] analysis shows that there is a significant positive correlation with physical surface hardness ($R^{2}=0.89$, *p* <0.01). This proves the feasibility of the rebound method of rapid screening of FCA concrete surface quality, provided that it is combined with an internal detection method to correct the error caused by local defects.

#### 3.5.3. Strength Prediction Correlation Modelling: Reliability Assessment of Multi-Parameter Fusion

To determine the optimal prediction framework, several candidate models, including single-variable linear and simple multi-linear regressions, were evaluated. In order to achieve accurate and non-destructive prediction of the compressive strength of FCA concrete, a multivariate nonlinear interaction regression model was created to take into account the UPV, the number of rebounds ($R_{n}$), and the Replacement Ratio ($R_{a}$). To accurately predict the compressive strength of FCA concrete non-destructively, a multivariate nonlinear interaction regression model was developed. While the mathematical structure of this model is adapted from established regression frameworks for recycled aggregates [75,85], the specific parameters and interaction terms were formulated to capture the synergy between internal defects and high replacement levels unique to FCA. The model is expressed as follows:

(2) $$\begin{array}{c} f_{cu}=\beta_{0}+\beta_{1}\cdot UPV+\beta_{2}\cdot R_{n}+\beta_{3}\cdot R_{a}+\beta_{4}(UPV{\cdot R}_{n})+\beta_{5}(UPV{\cdot R}_{a})+\varepsilon_{0} \end{array}$$

where $f_{cu}$ is the predicted compressive strength; $\beta_{0}$ is the intercept; $\beta_{1}$, $\beta_{2}$ and $\beta_{3}$ are linear sensitivity coefficients for UPV, $R_{n}$ and $R_{a}$ respectively; $\beta_{4}$ and $\beta_{5}$ are interaction coefficients representing the coupled effect of internal and surface properties; and $\varepsilon_{0}$ is the random error term.

**Figure 14:**
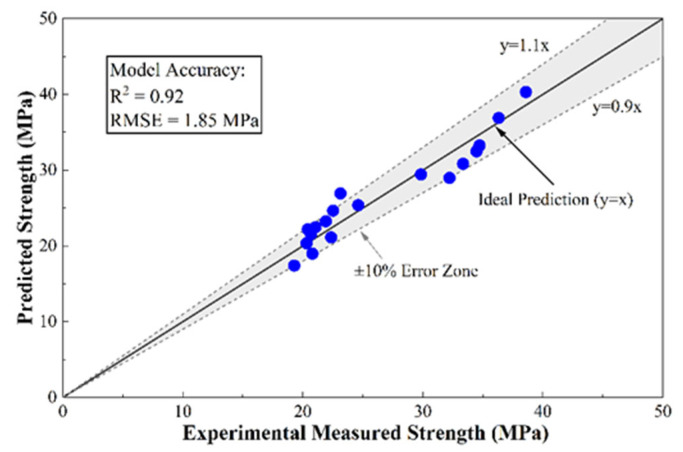
Comparison between experimental and predicted compressive strength using the multi-parameter regression model.

The scatter plot in Figure 14 demonstrates the model’s high fidelity, showing that the fit of the model is ($R^{2}$) > 0.85 and that the residual obeys the normal distribution, which is much greater than the single-parameter prediction equation.

In this model, $\beta_{1}$ to $\beta_{3}$ represent the linear sensitivity of strength to internal density (UPV), surface hardness ($R_{n}$) and ($R_{a}$) respectively. Terms $\beta_{4}$ and $\beta_{5}$ represent the physical basis of incorporating interaction terms in the shifting failure mechanism. At low $R_{a}$ values, UPV and $R_{n}$ contribute independently to strength; however, at high $R_{a}$ values, the porous structure of FCA causes a synergistic weakening of the matrix and ITZ [75]. In such cases, the impact of internal defects (reflected by UPV) on the carrying capacity is significantly amplified by the high replacement level, rendering simple linear models insufficient. Consequently, these interaction terms allow the model to capture the non-linear “curvature” of strength degradation as the aggregate crushing mode becomes dominant.

## 4. Discussion

The large-, medium- and small-scale performance evolution of concrete containing FCA is explored in detail, filling the gap between the macro-mechanical response and micro-evolution mechanism. Through theoretical analysis, combined with experimental evidence, the following clear conclusions and forward-looking research trajectory are drawn.

### 4.1. Mechanisms of Rheological and Mechanical Evolution

The present study found that fresh concrete has unique non-monotonic rheological characteristics that are determined by the competitive interaction of physical mechanisms. Under the medium–low-to-middle replacement system ($R_{a}\leq$ 60%), naturally enhanced mobility due to the low volume density of FCA appears [86]. However, when the threshold is exceeded, the capillary suction caused by the developed micropores of the aggregate becomes the dominant force, thereby reducing the effective water–binder ratio and leading to recession. Crucially, FCA’s “internal curing” function is recognised as an important buffer, and the release of moisture promotes continuous hydration in the ITZ. Under the hardening state, the mechanical integrity has a bifurcated degradation trajectory. Although the compressive strength begins to decline sharply once the replacement threshold exceeds 40%, the shear capacity exhibits even greater sensitivity to FCA inclusion. Specifically, the shear capacity undergoes a total reduction of 71.8%, falling from 13.1 kN in the control group to 3.7 kN at full replacement. The sharp decrease is related to the topological change of the failure mode [11]. Interface debonding, cross-particle fracture, etc., all show that the porous defects of the aggregate, itself, are the fundamental reason for the limited bearing capacity of the structure. The necessity of a non-linear model is further justified by the bifurcated degradation trajectory, which a constant-slope linear model cannot represent.

### 4.2. Strategic Optimisation and Engineering Thresholds

**Figure 15:**
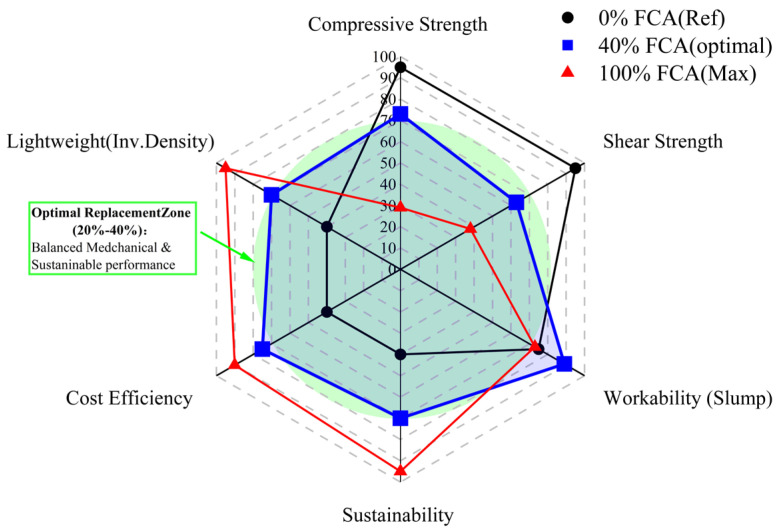
Comprehensive performance evaluation and optimisation of FCA concrete illustrating the optimal replacement zone (20–40%).

From a practical engineering perspective, the study takes the replacement interval of 20–40% as the best strategic window for FCA to utilize. This interval is defined by an integrated synergy of all investigated parameters, a balance clearly visualized by the radar chart in Figure 15. In this field, this material has reached an important equilibrium point, with good rheological properties and structural characteristics (28-day strength is 34.72 MPa), meeting the requirements of standard engineering. When the replacement rate is greater than 60%, non-compensatory intervention (interface reinforcement, gradient optimisation, etc.) cannot reduce potential security risks and can only be used directly [87].

### 4.3. Prospective Trajectories: From Micro-Mechanics to Digital Intelligence

In order to improve the elastic performance of the structure of recycled concrete in harsh environments, future surveys should go beyond the current macro-phenomenological description. High-fidelity characterisation methods such as synchronous accelerator micro-CT and nano-indentation should be used first to construct a conformational diagram including the porous-matrix internal stress concentration and dynamic crack propagation [88]. In addition, under severe exposure (freeze–thaw cycles, chemical erosion, etc.), there are still important cutting-edge research issues with respect to the effect of its internal curing ability to prolong its life. In parallel with scientific progress with respect to the above materials, the integration of digital intelligence gives us a transformative opportunity. The purpose of the future framework is to combine multi-source NDT analysis and deep learning algorithms to design an intelligent platform for whole-life-cycle health monitoring [89]. In essence, solving the technological problems of industrialization depends on the efforts of the whole society, adapting technical benchmarks at the macro level to encountered technical problems, and realising the deep integration of high-value waste and global sustainability tasks.

## 5. Conclusions

This study proposes an integrated multi-scale approach to the analysis of the rheological–mechanical transformation of concrete using FCA and outlines the controlling thresholds that restrict its engineering applicability. The main conclusions are summarized as follows:

1. FCA induces a strongly nonlinear response between fresh and hardened states. At low replacement levels, reduced density enhances flowability; at higher levels, micropores dominate capillary absorption, lowering the effective water-to-binder ratio and impairing rheology. In the hardened matrix, threshold-dependent degradation occurs, with strength loss and shear fragility increasing as replacement rises—reflecting a shift in dominant failure mechanisms from matrix continuity to interfacial debonding, particle fracture, and localized pore-controlled damage.
2. These findings define an optimal application window of 20–40%, where processability, mechanical reliability, and sustainability are effectively balanced. However, they also suggest that even high replacement ratios cannot be fundamentally offset by localized reinforcement alone.
3. This study establishes a multi-scale coupling mechanism between the microscopic pore characteristics and the macroscopic structural properties of waste FCA. Experimental results have shown that although FCA’s unique internal multi-scale pore network optimises the moist environment of the ITZ through the “internal curing” effect, it also becomes a stress concentration source that limits the structural load-bearing capacity. The high-precision, multi-parameter fusion nonlinear regression model (R^2^ > 0.85) constructed in this study achieves reliable and non-destructive evaluation of the strength of recycled concrete by integrating UPV, $R_{n}$ and $R_{a}$. This discovery provides a systematic theoretical framework and data support for rational design, performance optimisation and safety evaluation of waste foam concrete in high-value recycled building materials.

## Data Availability

The original contributions presented in the study are included in the article. Further inquiries can be directed to the corresponding author.
